# The Bioactive Potential of Trawl Discard: Case Study from a Crinoid Bed Off Blanes (North-Western Mediterranean)

**DOI:** 10.3390/md19020083

**Published:** 2021-02-02

**Authors:** Alfredo García-de-Vinuesa, Montserrat Demestre, Arnau Carreño, Josep Lloret

**Affiliations:** 1 Instituto de Ciencias del Mar, Centro Superior de Investigaciones Científicas (ICM, CSIC), Passeig Marítim de la Barceloneta, 37-49, E-08003 Barcelona, Spain; montse@icm.csic.es; 2 C/Maria Aurèlia Capmany 69, Institute of Aquatic Ecology, University of Girona, E-17003 Girona, Spain; arnau.carrenyo@udg.edu (A.C.); josep.lloret@udg.edu (J.L.)

**Keywords:** bioactive potential, discard, trawl, vulnerability, crinoid bed, sustainable exploitation

## Abstract

Although knowledge of the bioactive compounds produced by species inhabiting coastal waters is increasing, little is known about the bioactive potential produced by marine species occupying deeper habitats with high biodiversity and productivity. Here, we investigate about the bioactive potential of molecules produced by species that inhabit the crinoid beds, a poorly known essential fish habitat affected by trawling, wherein large amounts of commercial and noncommercial species are discarded. Based on a trawl survey conducted in 2019, 14% of the 64 species discarded on crinoid beds produce molecules with some type of bioactive potential, including; soft corals (*Alcyonium palmatum*); tunicates (*Ascidia mentula*); bony fish, such as horse mackerel (*Trachurus trachurus*); European hake (*Merluccius merluccius*); and chondrichthyans, such as small-spotted catshark (*Scyliorhinus canicula*). In addition, 16% of the discarded species had congeneric species that produce compounds with bioactive potential, indicating that such species might also possess similar types of bioactive molecules. Molecules with antioxidant, antitumour, antihypertensive, and antibacterial properties were the most frequent, which could provide the basis for future research aiming to discover new marine-based drugs and compounds for other human uses. Among all species or genera that produce compounds with bioactive potential, 68% presented medium or high vulnerability to trawling. Results show that the discarded catch contains many species, which produce different bioactive compounds that represent an added-value resource. These results highlight the importance of manage properly crinoid beds, to ensure that species that produce molecules with bioactive potential inhabiting these habitats are protected.

## 1. Introduction

In recent years, the study of molecules with bioactive potential produced by marine species has increased because of their possible uses in fields such as biomedicine, food technology, and pharmacology [1,2,3]. In the Mediterranean Sea, a high percentage of marine animals produce bioactive compounds. These animals could play a leading role in the discovery of future compounds and drugs of marine origin [4,5,6]. However, most studies of marine animals in the Mediterranean Sea have been conducted in shallow waters (above 50 m depth), whereas deeper waters have been neglected by comparison (below 100 m depth). A similar situation occurs worldwide: only a few studies, such as those of [7] and [8], which examined fishes collected at a 200 m depth and deep-sea fungi inhabiting sediment over 1000 m in depth, respectively, have screened organisms from deeper waters for bioactive potential. Consequently, the bioactive properties of compounds produced by species from deeper waters remains poorly known compared with species inhabiting shallow waters.

Crinoid beds are located in a transition zone between the end of a continental shelf and the beginning of a slope; because of the orography of this zone, there are currents that transport a large amount of nutrients that are harvested by phytoplankton, leading to high primary production [9,10,11]. This habitat is characterized by a high density of the crinoid *Leptometra phalangium*, which feeds on suspended matter and fixes a large amount of nutrients from the water column to the substrate [12,13]. Due to the unusually large amount of food available, several species with high commercial value, such as European hake (*Merluccius merluccius*) or monkfish (*Lophius budegassa* and *L. piscatorius*), occupy this habitat, which is also used by these species as a nursery area [14,15]. Despite its importance, this habitat does not have any special protections as other Mediterranean habitats, such as maerl beds or *Posidonia oceanica* meadows [16,17,18], both of which are included in Appendix A (Table A1) of the Habitats Directive of the European Union, so that currently crinoid beds are exploited by trawling, which may cause irreparable environmental damage [13,15,19,20].

One of the practices carried out by trawling that leads to resource waste and causes great damage to ecosystems is discarding, because the return to the sea of a fraction of the catch causes a loss of biodiversity and an alteration of the trophic chains in ecosystems, which negatively affects the stocks of commercial species [21,22]. For this reason, discard management is one of the main global challenges to improving the efficiency and sustainability of fisheries [23]. Trawling discards are mainly composed of commercial species that do not reach the legal minimum size and noncommercial species [24,25], and as in other habitats on the continental shelf, discards in crinoid habitats exceed the total commercial catch by more than half [26,27]. One of the major objectives of the European Union is the gradual implementation of a zero discard practice. Consequently, regulations have been implemented in recent years, such as the Landing Obligation (LO) [28], which oblige fishermen to land all species below the Minimum Conservation Reference Size (MCRS) and bans their sale. However, a large number of species do not have a MCRS, and as a result, this LO does not effectively prevent their discarding. Therefore, one of the strategies of the European Union to prevent the discarding of these species is to provide them with added value to increase their commercial interest. Thus, knowledge of the bioactive potential of the molecules produced by these species could be highly useful. However, to avoid causing more damage to the habitat, fisheries should be properly managed in a manner that fosters sustainable exploitation using knowledge of the conservation status of species, including the vulnerability of species to specific fishing methods [29,30].

Vulnerability is related to the likelihood that a species, population, community, or habitat will experience substantial alteration from short-term or chronic disturbance, and the likelihood that it would recover and in what time frame [31,32]. The factors that produce these alterations can be naturals or anthropogenic. In the specific context of the impact of trawling, species have specific biological traits that make them more or less vulnerable to such impacts, such as regeneration capacity, size, or reproductive frequency [33]. Thus, species with biological traits less vulnerable to trawling can better cope with this impact and show greater resilience. Consequently, their continued exploitation would become more sustainable compared with species showing high vulnerability to the impacts of trawling. Aside from the study of [6], no comprehensive studies to date have evaluated the vulnerability and conservation status of Mediterranean species that produce compounds with bioactive potential. However, such studies are critically important for understanding how these species cope with the impacts of human activity. Commercial fishing and other human activities affect marine species, which produce molecules with bioactive potential and can place them in danger of extinction [6].

The goal of this study was to identify species inhabiting crinoid beds, present in trawling discards, which produce compounds with bioactive potential and assess their vulnerability to trawling impacts. The bioactive properties of molecules produced by discarded species may represent an added-value fishery resource that is now being wasted. Our study aims to highlight the importance of these species for new human uses and the need to achieve sustainable exploitation of the habitats they inhabit.

## 2. Results

### 2.1. Data on Discards and Bioactives from Higher Taxa

An average of 53 kg of the catch was discarded per haul, which represents 45% of the total catch. The most abundant species found in the discard were *Leptometra phalangium*, *Alcyonium palmatum*, and *Funiculina quadrangularis*, while if we regard at biomass the most discarded species were *Scyliorhinus canicula*, *L. phalangium*, and *Diazona violacea*. A total of 64 species were identified in the discards, including 27 fish species and 37 invertebrate species, among which crustaceans and echinoderms were the most common with 10 and 11 species respectively (Table 1). Based on the relative frequencies, the highest percentage of species producing bioactive compounds among each taxon was Tunichata (25%), followed by Vertebrata (22.2%) and Cnidaria (20%). If only G2 (genera that produces compounds with bioactive potential) was considered, the highest relative frequency of taxa that produces bioactive molecules was Tunichata (50%), followed by Crustacea (20%) and Cnidaria (20%). Overall, 14.1% of the species of group G1 and 15.6% of the genera of group G2 were documented to have molecules with bioactive potential, whereas there were no studies relating to bioactive potential for the rest of the species (70.3% of the total).

### 2.2. Bioactive Compounds and Vulnerability of Discarded Species

The bioactive compounds most frequently found in the crinoid bed discard were antioxidant compounds, with 11 species producing them: six species from G1, including the shark *Scyliorhinus canicula* (30% of the total biomass discarded) and the bonyfish *Merluccius merluccius*, *Boops boops*, and *Trachurus trachurus* (0.1%, 6.2%, and 2.5% of the total biomass discarded, respectively), and five species from G2, including bony fish such as the anglerfish *Lophius piscatorius* and *L. budegassa*, or invertebrates such as the cephalopod *Sepia orbignyana* (Table 2). Of these aforementioned species, only *S. canicula* and *S. orbignyana* showed low vulnerability to trawling (the rest showed medium or high vulnerability to trawling). In addition, *S. canicula* possesses molecules with antitumor and antihypertensive properties, similar to *Raja clavata*, which is highly vulnerable to trawling. These two types of bioactive molecules were present in four other genera (*Pteria, Pagurus, Microcosmus*, and *Sepia*) and one species (*Ascidia mentula*). In addition, these five invertebrates taxa were the only ones with antibacterial potential and showed medium and low vulnerability to trawling. Molecules with cytotoxic activity have been observed in *Eledone cirrhosa* and *A. mentula*, which showed low and medium vulnerability to trawling, respectively, and in a species of the same genus of *Diazona violacea*, which had medium vulnerability and a discarded biomass close to 8%. *E. cirrhosa* also exhibited antifeedant properties similar to *Alcyonium palmatum*, which showed high vulnerability to trawling and was commonly discarded (5.8% of the total discard abundance). Other bioactive molecules were identified in a single species or genus. The genera *Astropecten* and *Sepia* possess molecules with antifouling and antifungal properties, respectively, and had low vulnerability to trawling. Anticoagulant molecules are present in *Microcosmus*, and anti-inflammatory properties are present in *Merluccius merluccius*, which showed high vulnerability to trawling.

## 3. Discussion

This study presents new information on the bioactive potential of compounds produced by species that are discarded by trawling in Mediterranean crinoid beds. Results show that the discarded catch contains many species that produce molecules with different bioactive properties that represent an added-value resource that is now being wasted, which could be especially valuable to treat different infectious diseases, certain cancer types and other illnesses. However, the bioactive potential of the molecules of many species inhabiting the crinoid beds may have remains unknown, and therefore further bioprospecting of discards is needed in order to develop commercially valuable products for pharmaceutical, agricultural, aquaculture, cosmetic, and other applications. Furthermore, this work shows the high vulnerability of many of these species to trawling impacts, highlighting the need of improving their management to achieve a balance between exploitation (i.e., for the discovery of new drugs) and the conservation of crinoid beds.

Overall, the outcomes of this study revealed that 30% of the taxa (species or genus) discarded are known to present compounds with bioactive potential. The goods and services that species inhabiting crinoid beds can offer in the form of molecules with properties beneficial for human health (e.g., antitumour, anti-inflammatory, etc.) and other human uses (e.g., antifouling properties) highlight the need for the habitats of vulnerable marine species that produce compounds with bioactive potential to be preserved [6]. The final management goal should be to find a balance between exploitation and conservation, which is needed not only for these particular species producing compounds with bioactive potential interest but also for all species inhabiting these habitats that are commercialized as seafood [27]. Previous studies have provided enlightening perspectives on how this balance could be achieved, including trawling within the exploitation of resources, as these studies have demonstrated that invertebrate species occupying these habitats, such as the echinoderms *Ophiura texturata* and *Echinus melo*, the sea squirt *Microcosmus sulcatus*, the cnidarian *Alcyonium palmatum*, or specifically the dominant species of the crinoid beds *Leptometra phalangium*, show high survival rates after being discarded [26,34].

The findings of this study demonstrate the additional potential economic value of discards through the bioactive potential of molecules that they produce. Adequate consideration of sustainability requires assessing the vulnerability of species producing these molecules to trawling (e.g., the Biological Trait Approach (BTA) used in this study), as uncontrolled exploitation by trawling often produces imbalances in populations as a result of biodiversity losses and fragmentation of the ecosystem itself [35,36,37,38]. BTA has been shown to be an effective tool for determining which species are suitable candidates for exploitation given its utility for assessing the vulnerability of species and marine communities to anthropogenic stressors such as trawling [29,39,40].

### 3.1. Species Producing Compounds with Bioactive Potential and High Vulnerability to Trawling

Several species that produce compounds with bioactive potential and high vulnerability to fishing were identified. For example, European hake (*Merluccius merluccius*) has molecules in its head oil with antioxidant and anti-inflammatory properties [41]. This species has high commercial value and is subject to strong fishing pressure in the Mediterranean Sea [42,43]. In addition, *M. merluccius* fall within the landing obligation (LO) (EU Reg. 1380/2013) and consequently, his discard is prohibited. Furthermore, these must be taken to ports and cannot be sold for economic benefit. Additionally, monkfish (*Lophius piscatorius* and *L. budegassa*) have high economic value, are high exploited, and are highly vulnerable to trawling. Peptides with antioxidant potential have been isolated from muscle of a species of the same genus, *Lophius litulon*. Although *Lophius* spp. is not affected by MCRS in the Mediterranean Sea they have a local minimum landing size in Catalonia [44]. Monkfish and European hake use crinoid beds as nursery areas. They are primarily discarded because they do not reach the legal minimum size [11,15,25]. The commercialization of undersized specimens is illegal, and their revaluation could lead to the creation of a black market that encourages fishermen to catch juveniles, which would only worsen the already poor state of conservation of their stocks in the Mediterranean [45].

Thornback rays (*Raja clavata*) also show high vulnerability to trawling and hydrolysates obtained from its muscle show antitumour, antioxidant, and antihypertensive properties [46]. Although this species does not have a legal minimum size either locally or at the European level, it should be protected because it is heavily exploited by trawling in the Western Mediterranean, where vulnerability assessments carried out with other methods indicate that this species also achieves a high vulnerability index and is classified on the IUCN Red List as a near-threatened species [47].

Other species that show high vulnerability to trawling include the bogue *Boops boops*, from which its lipid and protein fractions exhibit antioxidant properties [48], and the soft corals *Alcyonium palmatum* and *Pennatula rubra.* In addition these corals are considered by FAO as an indicator of the vulnerable marine ecosystem (VME), and *P. rubra* could be especially vulnerable to trawling due to only living in soft bottoms [32,49]. The discard of these three species primarily stems from their low or null economic value in the study area [27,34]. The bogue and the genus *Pennatula* have molecules with antioxidant properties, such as a briarane-type diterpenoid isolated from the methanolic extract of *P. aculeata*, with cyclooxygenase (COX) inhibitory activity [50,51], while *A. palmatum* hydrolizates have furanoesquiterpenes with antifeedant properties [3]. In addition, other species of the genus *Alcyonium* are known to produce molecules, such as sinularin or various terpenoids, with various bioactive qualities (antifungal, antimicrobial, antimicrobial, cytotoxic, anti-inflammatory, and antifoulant) [3]. Thus, an in-depth study of the species *A. palmatum* could provide insight into new bioactive molecules present in Mediterranean waters. The high vulnerability of these species to trawling, especially of the soft corals could finally put their populations in danger in the case of trawling exploitation [52,53]. However, these species could be used to identify potential molecules that, then, should be synthetized for human uses [6,54].

### 3.2. Species Producing Compounds with Bioactive Potential and Medium Vulnerability to Trawling

Species such as *Trachurus trachurus, Scorpaena notata*, and *Ascidia mentula* that presented intermediate vulnerability to trawling, showed antioxidant and antihypertensive qualities in the case of *T. trachurus* and fish protein hydrolysate in *S. notata*. The peptides isolated, mainly composed of hydrophilic amino acids, showed high antioxidative and angiotensin I converting enzyme inhibitory activities; and antibacterial and cytotoxic qualities in the case of *A. mentula* [55,56,57]. However, *T. trachurus* is discarded by small size (under MCRS) so its exploitation is illegal, and because of its low abundance and biomass, exploitation of *S notata* and *A. mentula* by trawling seems unrealistic. Other species with median vulnerability to trawling were the ascidians *Diazona violacea* and *Microcosmus sulcatus*. Some species with antitumour, cytotoxic, or antioxidant agents have been documented in these two genera, such as tanjungides A and B, two dibrominated indole enamides isolated from *D. violacea* or a sulfated polysaccharide, isolated from the ascidian *Microcosmus exasperatus*, which presents an anticoagulant effect in a dose-dependent manner through the inhibition of the intrinsic coagulation pathway [58,59]. For these reasons, these two species would make prime candidates for research on molecules with these specific bioactive potentials. However, achieving a balance between the exploitation and conservation of these moderately vulnerable species does not seem easy, particularly in slow-growing sessile species. On the other hand high survival to sessile invertebrates as genera *Microcosmus* sp. has been reported [26]. The exploitation of moderate vulnerable species could be carefully contemplated, although management measures such as the establishment of Fisheries Restricted Areas could be successful in achieving a balance between exploitation and conservation of these species [60,61].

### 3.3. Species Producing Compounds with Bioactive Potential and Low Vulnerability to Trawling

Other species discarded in crinoid beds have low vulnerability to trawling. This is the case for *Scyliorhinus canicula*, which has molecules with antitumour, antioxidant, and antihypertensive qualities that can be used to discover new medicines against cancer and coronary diseases, such as two peptides that were isolated from the epigonal tissue of this species, which exhibit antineoplastic activity on the human prostate and breast cancer cells in vitro [62,63]. The exploitation of *S. canicula*, which is considered a lower-risk species in terms of its conservation status and has a high abundance in the Mediterranean [64], by the trawling fleet might not cause significant damage to its population through controlled management. Other species that also show low vulnerability to trawling are *Pagurus excavatus*, *Sepia orbignyana*, and *Astropecten irregularis*, but the bibliographic search shows that molecules responsible of their bioactive potential remain unknown, whereas other species of their genus have molecules with some type of bioactive potential, such as *S. officinalis*, with antibacterial properties in their ink and internal skeleton [65,66], and other antifungal and antitumour properties attributed to their salivary glands; *Pagurus bernhardus*, which several tissues, hemolymph and hemocyte extract exhibit antibacterial activity against several species of bacteria strains; or *Astropecten articulatus*, which produces antifouling compounds [1,67,68].

### 3.4. Limits to the Applicability of the Results

The methodology and results of this study could be used to discard species in other crinoid beds that are home to rich and diverse fish and invertebrate communities [11,13,69]. Additionally, these results could be applied to discard vulnerable habitats for which there is a need to find a balance between exploitation and conservation, such as muddy habitats on the continental shelf, which can also be highly diverse and productive [70,71]. However, this study has some limitations that need to be addressed in future research. No studies relating to the bioactive potential were detected for 70% of the species analyzed in this work. Therefore, new studies for assessing the possible bioactive potential of compounds produced by these species are needed, given that these species could provide goods and services to humans that have yet to be identified.

## 4. Materials and Methods

### 4.1. Study Area and Analysis of the Discards

**Figure 1:**
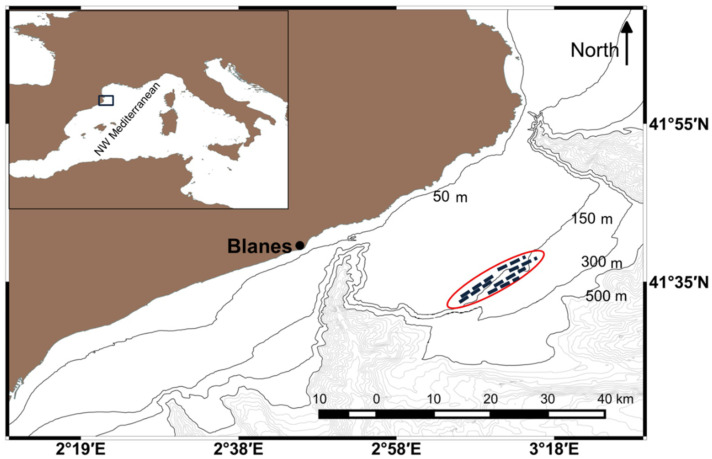
The study area. The red ellipse shows the fishing ground where the crinoid bed is located; the 6 hauls of trawling (indicated by the broken lines) were carried out in 2019.

The study area was located in the Northwestern Mediterranean in a trawl fishing ground adjacent to Blanes port (Figure 1), which features a crinoid bed habitat [25,26].

On the crinoid bed, six trawl hauls were carried out in June 2019 onboard a commercial trawl fishing vessel at a depth between 113 and 172 m. Normal fishing hauls was carried out with classic trawl gear with a mesh size of 40 mm square mesh codend. The time of each haul was 120 min. Between 7 and 10 kg of subsample was taken from the discard in each of the six hauls. These samples were transferred to the laboratories of the Institute of Marine Sciences (ICM-CSIC), where the discard was classified at the species level. The abundance and biomass data were obtained by species and extrapolated to the total discard of each haul. After, the mean percentage per species of abundance and biomass was calculated.

### 4.2. Bioactive Potential of Compounds Produced by the Species

The bioactive potential produced by species identified in the discards was gathered from the literature following the methodology as suggested by Carreño and Lloret [6]. The PubMed, SciencieDirect, and PLOS ONE databases were used, and the search criteria used included the name of the species or its genus + the type of bioactive potential (antitumour, antibacterial, antifeedant, cytotoxic, antifungal, antifoulant, antihypertensive, antioxidative, anti-inflammatory, or anticoagulant).

According to the results of the bibliographic search, the discarded species were classified into three groups according to the typology of bioactive potential produced:

(G1) Species that produce compounds with bioactive potential—there is evidence in the literature that indicates that the species produces a molecule with bioactive potential.

(G2) Genus that produces compounds with bioactive potential—in cases where we could not find any information for G1, the bioactive potential produced by the genus to which the species belonged was searched; hence, G2 indicates that other species in the genus produce compounds with bioactive potential according to the literature.

(G3) No proven production of compounds with bioactive potential—there are no studies to date that indicate that the species or genus produce any kind of molecule with bioactive potential.

The species were then classified by higher taxa (Vertebrata, Tunichata, Cnidaria, Mollusca, Crustacea, and Echinodermata) according to the former three categories G1, G2, and G3. Finally, the type of bioactive potential for each species was indicated.

### 4.3. Vulnerability of the Species to Trawling

A biological trait approach (BTA) [72,73,74] was developed for the species, which produce compounds with bioactive potential. BTA is a tool that has been shown to be useful for studying the vulnerability of marine species to anthropogenic factors, such as trawl impact. The BTA classifies the vulnerability of species by taking into account biological traits that are closely related to the impact to which they are exposed. The BTA developed in this study considered 11 biological traits (BTs) with high sensitivity to trawling disturbance. BTs were classified into 3 classes, each one assigned a specific weight according to its relevance to vulnerability to trawling [30] (Table 3). The first survival class (weight = 0.5) contained BT regeneration, fragility, protective structure, and resistance to air exposure; the second was the catchability (weight = 1) class and contained BT mobility, size, sociability, and environmental position; and the third was the resilience class (weight = 1.5) and contained BT age at maturity, reproductive frequency, and egg development. Each biological trait (BT) was split into 2 or 3 categories, and each trait was assigned a score depending on the trawling sensitivity using an ordinal scale ranging from 1 to 3 (1 = low sensitivity, 2 = moderately sensitive, and 3 = highly sensitive). To assess species vulnerability, each species was assigned a category for each BT, and the score for each category was multiplied by the specific weight of its class. Finally, the scores obtained from this process for each species were added to obtain a total score of between 15 and 21 points, from which 3 ranges of vulnerability to trawling were established (low = (15–17); medium = (17–19); and high = (19–21)). The assignment of categories by species was carried out using expert knowledge and information codified in the biological databases MarLIN (https://www.marlin.ac.uk/species), FishBase (http://www.fishbase.org/), and WorMs (http://www.marinespecies.org/). In addition, this information was complemented by a literature review of studies conducted in the Mediterranean.

## 5. Conclusions

The results indicate that animals inhabiting relatively deep Mediterranean habitats, such as crinoid beds found between the end of continental shelf and the start of the slope, produce molecules with bioactive potential that could provide an excellent resource for the discovery of new marine drugs and for other human uses. These new resources, which are now being thrown away or dumped at sea as part of the fisheries’ discards, can provide an added value to goods and services that this poorly known marine habitat provides. Furthermore, the fact that many of these species are high or medium vulnerable to fishing highlights the need to develop proper management and conservation strategies of marine habitats that consider more than just fisheries and that encompass sustainable marine biotechnology. Thus, the results indicate the need for introducing specific measures that allow species that produce compounds with bioactive potential (including those that are currently only potential sources of molecules with pharmacological properties beneficial for human health) to be exploited in a sustainable manner. More research is needed on marine bioprospecting of deep habitats, as the vast majority of the species discarded in crinoid beds lack indication of secretions with biotechnological potential. However, it is necessary to emphasize that unregulated exploitation of highly vulnerable species by trawling to obtain molecules with bioactive potential could endanger their populations. There is thus a need for the marine biotechnology industry to ensure that in vitro or recombinant options are produced after isolation of bioactive compounds from the marine species so that the production of these compounds (after their discovery in the sea) continues in the laboratory and is not dependent on the collection of wild animals.

## Figures and Tables

**Table 1:** Bioactive potential produced by higher taxa. NS is the number of species found in the discard; G1: species with bioactive potential, G2: genus that produces compounds with bioactive potential and G3: species and genus without demonstrated production of molecules with bioactive potential; %G1, %G2 and %G3 are the relative frequencies of groups G1, G2 and G3, respectively.

Taxa	NS	G1	G2	G3	% G1	% G2	% G3
Vertebrata	27	6	3	18	22.2	11.1	66.7
Tunichata	4	1	2	1	25	50	25
Cnidaria	5	1	1	3	20	20	60
Mollusca	7	1	1	5	14.3	14.3	71.4
Crustacea	10	0	2	8	0	20	80
Echinodermata	11	0	1	10	0	9.1	90.9
TOTAL	64	9	10	45	14.1	15.6	70.3

**Table 2:** Species that produce compounds with bioactive potential (G1) and genera that produce molecules with bioactive potential (G2) found in the discard of trawling in crinoid beds. The table shows ordered (by species) from highest to lowest abundance of types of bioactive molecules, the vulnerability range (vul.rank), percentage of abundance (Ab. (%)) and biomass Bio. (%), and the type of bioactive potential (Bio. Pot.) indicated by codes: Hy (antihypertensive), Ox (antioxidative), Fe (antifeedant), In (anti-inflammatory), Ci (cytotoxic), Tu (antitumoral), Ba (antibacterial), Co (anticoagulant), Fo (antifoulants), and Fu (antifungal).

Species	G1	G2	Bio. Pot.	Vul. Rank	Ab. (%)	Bio. (%)
*Sepia orbignyana*		x	Ba, Ox, Tu, Fu	Low	0.04	0.01
*Scyliorhinus canicula*	x		Ox, Hy, Tu	Low	2.70	29.88
*Raja clavata*	x		Ox, Tu, Hy	High	0.04	3.16
*Microcosmus sulcatus*		x	Ba, Tu, Co	Medium	0.04	0.36
*Trachurus trachurus*	x		Hy, Ox	Medium	3.05	2.49
*Merluccius merluccius*	x		In, Ox	High	0.13	0.12
*Diazona violacea*		x	Ci, Tu	Medium	0.49	7.98
*Scorpaena loppei*		x	Ox, Hy	Medium	0.09	0.11
*Scorpaena notata*	x		Ox, Hy	Medium	0.04	0.05
*Ascidia mentula*	x		Ba, Ci	Medium	0.09	0.06
*Eledone cirrhosa*	x		Ci, Fe	Low	0.04	0.07
*Alcyonium palmatum*	x		Fe	High	5.83	4.51
*Boops boops*	x		Ox	High	0.57	6.21
*Lophius budegassa*		x	Ox	High	0.44	0.73
*Lophius piscatorius*		x	Ox	High	0.22	0.69
*Pennatula rubra*		x	Ox	High	0.04	0.03
*Pteria hirundo*		x	Ba	Low	0.22	0.07
*Astropecten irregularis*		x	Fo	Low	0.57	0.12
*Pagurus excavatus*		x	Ba	Low	0.09	0.13

**Table 3:** Biological traits, category scores of categories, and each class with its weight used to determine the vulnerability of the species to trawling.

Biological Trait	Category	Score	Class	Weight
Regeneration	Yes	1	Survival	0.5
	No	2		
Fragility	Low	1	Survival	
	Medium	2		
	High	3		
Protective structure	Yes	1	Survival	
	No	2		
Resistance to air exposure	Low	3	Survival	
	Medium	2		
	High	1		
Mobility	Sessile or sedentary	3	Catchability	1
	Crawler	2		
	Swimmer	1		
Size	Small	1	Catchability	
	Medium	2		
	Large	3		
Sociability	Schools	3	Catchability	
	Small groups	2		
	Solitary	1		
Environmental position	Benthic	3	Catchability	
	Demersal	1		
Age at maturity	>1 year	2	Resilience	1.5
	<1 year	1		
Reproductive frequency	Annual	2	Resilience	
	More than annual	1		
Egg development	Pelagic	1	Resilience	
	Benthic	2		

## Data Availability

Data is contained within the article.

## Appendix A

**Table A1:** Discard abundance and biomass by species expressed in individuals per 2 h (Ind/2 h), in reference to haul time (2 h), and in grams (g) respectively. Additionally, it can see abundance and biomass per species expressed in percentage (%).

Species	Abundance (Ind/2 h)	Biomass (g)	Abundance (%)	Biomass (%)
*Alcyonium palmatum*	372.712	1956.323	5.830	4.510
*Anseropoda placenta*	0.166	1.174	0.003	0.003
*Argentina sphyraena*	7.428	50.041	0.116	0.123
*Arnoglossus laterna*	12.270	105.770	0.192	0.260
*Arnoglossus ruepelli*	1.326	10.436	0.021	0.026
*Ascidia mentula*	5.754	24.403	0.090	0.060
*Ascidia sp. 1*	2.283	12.254	0.036	0.030
*Astropartus mediterraneus*	0.166	2.218	0.003	0.005
*Astropecten irregularis*	36.440	48.806	0.570	0.120
*Boops boops*	36.440	2525.731	0.570	6.210
*Calliactis parasitica*	5.273	6.210	0.082	0.015
*Callionymus maculatus*	4.444	11.576	0.070	0.028
*Capros aper*	71.962	384.181	1.126	0.945
*Cidaris cidaris*	1.326	10.436	0.021	0.026
*Dardanus arrosor*	15.653	167.735	0.245	0.412
*Diazona violacea*	31.326	3245.626	0.490	7.980
*Echinaster sepositus*	0.663	5.218	0.010	0.013
*Echinus melo*	2.520	122.468	0.039	0.301
*Eledone cirrhosa*	2.557	28.470	0.040	0.070
*Eutrigla gurnardus*	5.903	92.882	0.092	0.228
*Funiculina quadrangularis*	209.817	104.101	3.282	0.256
*Gracilechinus acutus*	2.487	110.884	0.039	0.273
*Helicolenus dactylopterus*	32.035	548.680	0.501	1.349
*Illex coindetii*	2.951	66.875	0.046	0.164
*Inachus dorsettensis*	3.946	13.350	0.062	0.033
*Lepidorhombus boscii*	85.724	1164.882	1.341	2.864
*Lepidotrigla cavillone*	8.854	30.500	0.138	0.075
*Leptometra phalangium*	4561.930	6500.935	71.322	15.948
*Liocarcinus depurator*	5.372	60.138	0.084	0.148
*Lophius budegassa*	28.129	296.906	0.440	0.730
*Lophius piscatorius*	14.065	280.637	0.220	0.690
*Macropipus tuberculatus*	3.814	28.178	0.060	0.069
*Macropodia longipes*	9.650	19.740	0.151	0.049
*Macroramphosus scolopax*	130.228	1385.659	2.037	3.407
*Merluccius merluccius*	8.311	48.806	0.130	0.120
*Microcosmus sulcatus*	2.557	146.419	0.040	0.360
*Mullus barbatus*	2.951	139.323	0.046	0.343
*Munida intermedia*	7.893	29.495	0.123	0.073
*Nemertesia ramosa*	71.995	446.667	1.126	1.098
*Ophidiaster ophidiamus*	0.166	0.522	0.003	0.001
*Ophiura texturata*	31.338	186.259	0.490	0.458
*Pagellus bogaraveo*	27.923	1180.589	0.437	2.903
*Pagellus erythrinus*	3.979	129.408	0.062	0.318
*Pagurus excavatus*	5.754	52.874	0.090	0.130
*Parthenope macrochelos*	0.332	6.001	0.005	0.015
*Pennatula rubra*	2.557	12.202	0.040	0.030
*Phycis blennoides*	5.903	162.543	0.092	0.400
*Pisa armata*	0.332	2.152	0.005	0.005
*Pteria hirundo*	14.065	28.470	0.220	0.070
*Raja clavata*	2.557	1285.235	0.040	3.160
*Rossia macrosoma*	20.693	615.837	0.324	1.514
*Scorpaena loppei*	5.754	44.739	0.090	0.110
*Scorpaena notata*	2.557	20.336	0.040	0.050
*Scyliorhinus canicula*	172.611	12,152.794	2.700	29.880
*Scyliorhinus canicula* (eggs)	34.356	55.964	0.537	0.138
*Sepia orbignyana*	2.723	4.067	0.043	0.010
*Sepietta oweniana*	1.326	8.349	0.021	0.021
*Serranus hepatus*	2.951	2.229	0.046	0.005
*Spatangus purpureus*	33.262	2321.766	0.520	5.709
*Spicara maena*	1.326	41.745	0.021	0.103
*Squilla desmaresti*	1.161	2.904	0.018	0.007
*Todaropsis eblanae*	1.161	10.958	0.018	0.027
*Torpedo marmorata*	4.278	995.869	0.067	2.449
*Trachurus trachurus*	194.987	1012.733	3.050	2.490
*Trisopterus capellanus*	11.911	116.107	0.186	0.285
Total	6395.283	40,686.784	100.000	100.000
Organic matter and detritus (OMD)		12,320.00		
Total + OMD		53,006.784		
